# Intrarenal Pressure State of the Art: The Past, Present and Future

**DOI:** 10.3390/jcm15218756

**Published:** 2026-11-09

**Authors:** Maanya Bhardwaj, Oliver Wiseman

**Affiliations:** Department of Urology, Addenbrooke’s Hospital, Cambridge University Hospitals NHS Foundation Trust, Cambridge CB2 0QQ, UK; maanyab@gmail.com

**Keywords:** intrarenal pressure, intrarenal backflow, retrograde intrarenal surgery, stone surgery, kidney stones, ureteroscopy, flexible and navigable suction ureteral access sheaths, endourology

## Abstract

**Background:** Kidney stone disease is increasingly prevalent worldwide, resulting in a growing use of ureteroscopy (URS) for stone management. Although URS is generally safe and effective, elevated intrarenal pressure (IRP) during the procedure may contribute to perioperative morbidity, particularly infectious complications. Excessive IRP can promote intrarenal backflow into tubular, venous, lymphatic, and interstitial compartments, potentially resulting in tissue injury, impaired renal perfusion, and systemic exposure to bacteria and endotoxins. Advances in pressure-monitoring technology have enabled continuous intraoperative assessment of IRP and highlighted substantial pressure variability and transient spikes during routine URS. **Methods:** This narrative review examines the historical understanding and contemporary evidence surrounding IRP during URS, based on a structured search of PubMed/MEDLINE, the Cochrane Library and Europe PMC (January 1980 to October 2026), supplemented by manufacturer and regulatory sources. The review evaluates current approaches to IRP measurement, the pathophysiological and clinical consequences of elevated pressure, patient-specific susceptibility, and strategies aimed at pressure mitigation. **Results:** Available experimental and clinical evidence indicates that elevated IRP may contribute to acute renal tissue injury, impaired renal perfusion and intrarenal backflow, although most reported pressure thresholds derive from experimental models rather than validated human safety limits. IRP is influenced by irrigation inflow, outflow resistance, and renal compliance, and may vary considerably during URS, with transient pressure spikes occurring even during routine procedures. Multiple strategies can reduce IRP. Novel pressure-guided platforms integrating real-time pressure monitoring with automated irrigation and suction control can regulate IRP in early studies, but evidence that they improve clinical outcomes remains limited. Suction-assisted access sheaths are associated with fewer postoperative infectious complications in comparative studies, although this benefit has not been shown to result specifically from IRP reduction. **Conclusions:** IRP is an increasingly recognised and potentially modifiable determinant of safety during ureteroscopy. Further clinical research, including standardised reporting of IRP metrics, is required to establish clinically relevant pressure thresholds and determine whether pressure-guided URS improves patient outcomes. The integration of real-time monitoring with automated irrigation and suction control represents a promising direction toward safer, pressure-conscious endourological practice.

## 1. Introduction

Kidney stone disease is a common and growing global health problem, with an estimated 106 million incident cases worldwide in 2021 and a prevalence of approximately 11% among adults in the United States, creating a major clinical, economic and quality-of-life burden for patients and healthcare systems [1,2,3]. Recurrence rates are high, with up to 50% of individuals who have had stone disease experiencing a second stone within 10 years [4].

The increasing prevalence of stone disease has a direct effect on healthcare resources, with a sustained increase in the number of ureteroscopy (URS) procedures performed in England over the last two decades [5,6]. Technological advances in the design and size of ureteroscopes have enabled easier access to the upper urinary tract, avoiding the need for surgical incisions. Furthermore, high-quality digital optics provide accurate assessment of stone size and location. URS is a safe procedure, although complications do occur; reported rates include fever or urinary tract infection in 0.2–15%, urosepsis in 0.1–4.3%, ureteral perforation in 0.3–7.4% and ureteral avulsion in 0.04–0.9% of procedures [7]. Other complications include haematuria, ureteral stricture and persistent vesicoureteric reflux [7,8]. Sepsis is considered the most dangerous complication in URS as it is associated with high morbidity and mortality [9,10].

There is increasing focus on intrarenal pressure (IRP) management during URS. IRP refers to the hydrostatic pressure within the renal collecting system and renal parenchyma [11]. It is commonly estimated by renal pelvic pressure and reflects pressure transmission to intrarenal tubular, interstitial, venous and lymphatic compartments [12]. As direct measurement of intraparenchymal pressure is rarely feasible in clinical practice, renal pelvic pressure is commonly used as a surrogate measure of IRP [12,13,14]. Flow imbalance arising from excessive irrigation inflow compared to insufficient outflow can result in significant increases in IRP [13]. Balance between clear intraoperative vision, procedure time and patient safety via adequate, but not excessive, irrigation needs to be achieved [14,15]. The link between increases in IRP and septicaemia is poorly understood, although it is believed that intrarenal backflow (IRB) of urine and irrigation fluid from the collecting system to the tubules, venous and lymphatic system predisposes to sepsis [15].

This review aims to summarise the historical understanding of IRP, describe current measurement techniques, the consequences of increased IRP, patient-specific susceptibility and mitigation strategies, and summarise where IRP measurement will fit into the workflow of ureteroscopy cases.

## 2. Materials and Methods

This is a narrative (non-systematic) review. We searched PubMed/MEDLINE, the Cochrane Library and Europe PMC for English-language articles published between January 1980 and October 2026, using combinations of the terms intrarenal pressure, renal pelvic pressure, intrapelvic pressure, intrarenal backflow, pyelovenous backflow, ureteroscopy, retrograde intrarenal surgery, irrigation, ureteral access sheath, suction, flexible and navigable suction ureteral access sheath and pressure monitoring. We prioritised human clinical studies, systematic reviews and consensus statements, and drew additionally on experimental (in vitro, ex vivo and animal) studies for physiological and mechanistic data. Because pressure-sensing and fluid-management technologies are developing rapidly, peer-reviewed conference abstracts, manufacturer information and United States Food and Drug Administration (FDA) 510(k) records were also consulted for device-specific and regulatory information. Reference lists of key papers and recent reviews were hand-searched for further sources. The review was prepared with reference to the Scale for the Assessment of Narrative Review Articles (SANRA) [16].

Studies were selected on the basis of relevance to the physiology, measurement, clinical consequences and mitigation of IRP during ureteroscopy, rather than by formal quality scoring, and no quantitative synthesis was performed. Where possible, the type of supporting evidence (experimental, clinical association or expert consensus) is stated explicitly. Pressures are reported in mmHg with the equivalent value in cmH_2_O (1 mmHg ≈ 1.36 cmH_2_O) to facilitate comparison between studies.

**Figure 1:**
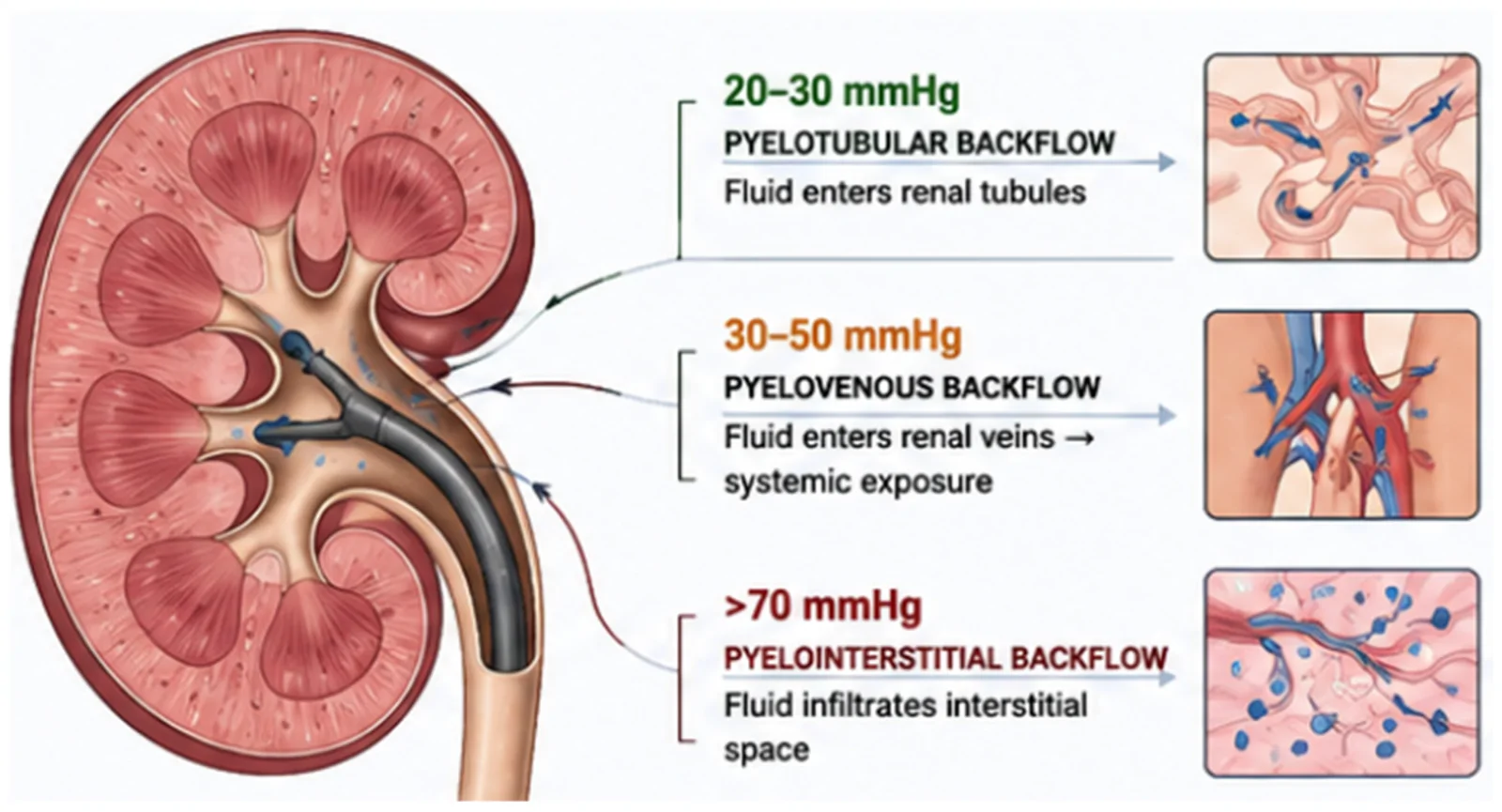
Schematic representation of IRP and IRB pathways. The pressure ranges and backflow pathways shown are derived predominantly from experimental and animal studies and do not represent validated human clinical thresholds [15,18]. Figure generated with AI assistance (Chat GPT 5.5) [17].

**Figure 2:**
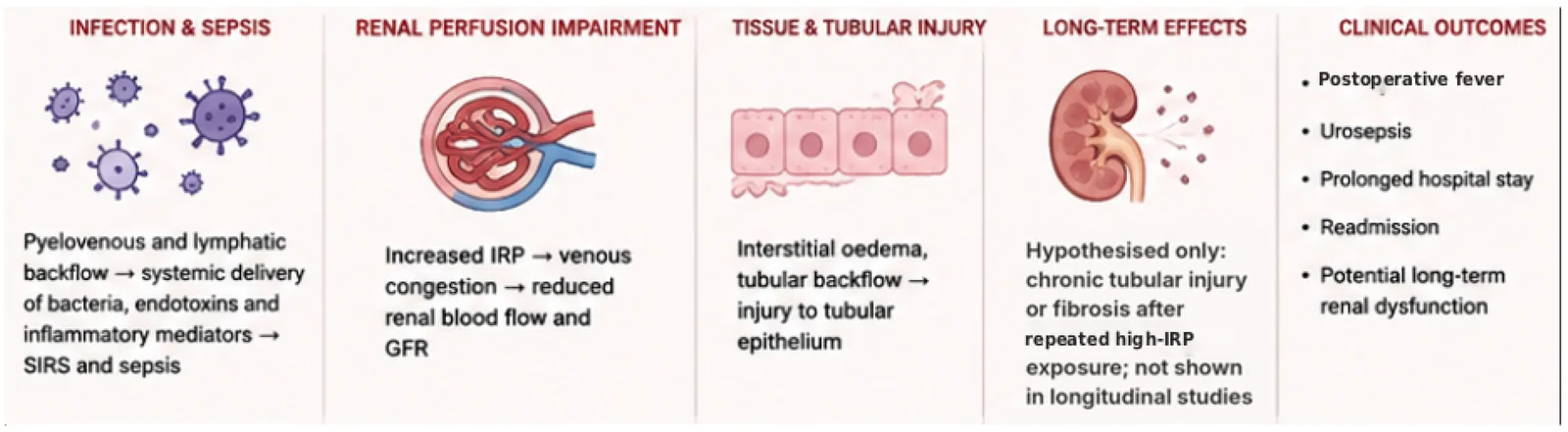
Schematic representation of the potential consequences of elevated IRP. Infectious complications and sepsis are supported by observational human clinical evidence; impaired renal perfusion and acute tissue injury are derived predominantly from experimental and animal models, whereas chronic tubular injury or fibrosis is hypothesised and has not been demonstrated in longitudinal studies [18,19,20]. Figure generated with AI assistance (Chat GPT 5.5) [17].

During the preparation of this work, the authors used a large language model, Claude (Anthropic), to assist with structuring, language editing, formatting and reference checking, and ChatGPT (GPT-5.5; OpenAI) to generate the schematic illustrations in Figure 1 and Figure 2 [17]. The authors reviewed and edited the content and take full responsibility for the publication.

## 3. The Past: Early Understanding of Intrarenal Pressure

IRP increases during URS, typically exceeding physiological levels (0–15 mmHg; 0–20 cmH_2_O) [11,15]. IRP depends on the balance between irrigation inflow and outflow, and on the compliance of the renal collecting system [15]. Raised IRP leads to an increase in IRB, with IRB thresholds varying according to papillary morphology, starting as low as 20 mmHg (27 cmH_2_O) and progressing at higher pressures [15]. Largely on the basis of experimental and animal data, IRB has been categorised as follows:

- 20–30 mmHg (27–41 cmH_2_O): pyelotubular backflow, whereby fluid enters the renal tubules [15].
- 30–50 mmHg (41–68 cmH_2_O): pyelovenous backflow, whereby fluid enters the renal veins, leading to systemic exposure [15].
- >70 mmHg (>95 cmH_2_O): pyelointerstitial backflow, whereby fluid infiltrates the renal interstitial tissue [15].

IRB is both pressure- and time-dependent. Animal studies using dynamic magnetic resonance imaging demonstrated spreading of IRB throughout the renal cortex when IRP was sustained at 20–70 mmHg (27–95 cmH_2_O) for 30–60 min, and IRB was documented at IRP as low as 20 mmHg (27 cmH_2_O) [18]. These ranges derive predominantly from experimental and animal models and should not be interpreted as validated human clinical safety thresholds; susceptibility to IRB is modified by papillary morphology, renal compliance, obstruction, infection and duration of exposure (Section 4.4). A Delphi consensus of endourologists did not identify an evidence-based IRP threshold, although initial concern was expressed at approximately 40 cmH_2_O (29 mmHg) [11]. Reported pressure values are summarised in Table 1, which distinguishes experimental backflow thresholds, irrigation pressure settings applied at the irrigation source and intraoperative clinical measurements, and states the type of supporting evidence for each.

Renal compliance measures how easily the system responds to increases in pressure and can expand in response to rising pressure. It is believed that the cone-shaped morphology of the renal papilla provides protection against intrarenal reflux by compressing the ducts of Bellini when pressure increases around the papilla [13,18,21,26]. Experimental studies have shown that flattened papillae, noted in conditions such as medullary sponge kidney, compound papillae, and papillary pitting, compromise these anti-reflux mechanisms [18,21,26]. Hydronephrosis also predisposes to increased IRP due to papillary thinning and flattening caused by prolonged elevated pressure in the kidney [13,18]. Since medullary papillae play a major role in protection against reflux, flattening of these papillae increases susceptibility to elevated IRP. Moreover, experimental results indicate that renal resilience to IRP is significantly lower under hypotensive conditions such as sepsis [10,18,27]. These physiological insights have become clinically relevant with the widespread adoption of endourological interventions.

## 4. The Present: Intrarenal Pressure in Modern Endourology

### 4.1. Sources of Elevated Intrarenal Pressure

As URS has evolved in complexity and frequency, understanding how modern instrumentation and technique influence IRP has become increasingly important. URS inherently predisposes to elevated IRP due to the use of irrigation in a relatively closed system [28]. During flexible ureteroscopy, continuous irrigation is required to maintain visibility and permit laser lithotripsy. However, the narrow calibre of the ureter and intermittent obstruction of outflow by instruments or stone fragments can convert the collecting system into a partially closed hydraulic environment, predisposing to rapid pressure elevation [28]. Irrigation-related pressure rises are influenced by irrigation modality (gravity, hand-held, or pressurised systems), scope diameter, aspiration-channel obstruction, use of an access sheath, the scope-to-sheath diameter difference, stone burden and operative duration [29,30]. By contrast, obstruction of the working channel by instruments predominantly reduces inflow rather than outflow, unless direct in-scope suction is used [29,30]. Transient but repetitive pressure spikes frequently occur during use of handheld on-demand irrigation systems, laser lithotripsy, basketing and scope deflection, particularly when outflow is restricted [20,30].

Contemporary flexible and navigable suction ureteral access sheaths (FANS) were developed to address this imbalance by facilitating continuous egress of irrigation fluid, stone fragments and intrarenal debris [31]. However, when suction and irrigation are manually controlled, IRP regulation remains operator-dependent and susceptible to variability, particularly during complex or prolonged procedures [30,31].

### 4.2. Measurement of Intrarenal Pressure

Historically, IRP has been assessed using nephrostomy-based pressure transducers or retrograde ureteral catheters. Although these methods provided important physiological insights, their clinical applicability has been limited by invasiveness, inability to provide continuous intraoperative feedback, and poor correlation between irrigation bag pressure and true renal pelvic pressure [32].

The first platform cleared by the FDA for intraoperative IRP monitoring during ureteroscopy was the LithoVue Elite™ digital flexible ureteroscope (Boston Scientific, Marlborough, MA, USA; 510(k) K221515, 2023), which incorporates a pressure sensor at the tip of the ureteroscope [30,33]. Early clinical and experimental work by Bhojani, Chew and colleagues demonstrated the feasibility of continuous pressure acquisition during ureteroscopy and highlighted the discrepancy between irrigation system pressure and true intrarenal pressure dynamics [30,34]. Importantly, scope-integrated monitoring enabled pressure visualisation without requiring additional access devices and represented a major transition from surrogate estimation toward direct physiological monitoring of IRP during ureteroscopy. Intravascular pressure guidewires placed within the collecting system offer an alternative research tool for continuous IRP measurement and have been used in pilot and observational clinical studies [20,35].

IRP is not uniformly distributed throughout the collecting system, irrespective of whether a UAS is used. In a human study measuring pressure in 45 calyces of eight patients, Patel and colleagues found that intracalyceal pressure frequently differed from renal pelvic pressure; with a UAS in place, mean interpolar calyceal pressure was 30.8 mmHg (41.9 cmH_2_O) compared with 17.9 mmHg (24.3 cmH_2_O) in the renal pelvis, and lower-pole pressure was strongly influenced by sheath size [24]. Ex vivo work using a scope-integrated sensor similarly demonstrated significant differences between calyceal and pelvic pressures across irrigation settings and sheath sizes [36]. Pressure measured at a single point, whether at the ureteroscope tip, within a sheath or in the renal pelvis, may therefore not represent pressure elsewhere in the kidney. This has implications for the interpretation of pressure readings and for comparison between studies that use different sensor locations [11,24].

More recently, pressure-monitoring technology has been integrated into FANS, allowing simultaneous pressure sensing, irrigation regulation and suction-assisted outflow [25,37,38]. Two emerging systems include the i-MIMERsys device (ZSR Biomedical Technology Co., Ltd., Dongguan, Guangdong, China) and the Tidor platform, both of which aim to facilitate continuous real-time IRP monitoring while improving intrarenal fluid management during flexible ureteroscopy [39].

The i-MIMERsys system integrates pressure sensing within a flexible and navigable suction UAS combined with an autoregulation platform capable of dynamically adjusting irrigation and suction to maintain IRP within predefined thresholds [39]. In an in vitro comparison against a urodynamic reference, i-MIMERsys showed a mean difference of 0.96 ± 2.26 mmHg (intraclass correlation coefficient 0.955) [39]. Separately, a preclinical animal study and pilot clinical study demonstrated the feasibility of continuous IRP monitoring via a pressure-sensing FANS during retrograde intrarenal surgery [37].

The Tidor platform (YiGao Medical Technology Co., Ltd., Hangzhou, Zhejiang, China) similarly incorporates continuous pressure monitoring within a suction-assisted access system but differs in its technical approach to pressure regulation and fluid evacuation [39]. In the same in vitro comparison, Tidor showed a mean difference of 1.09 ± 2.57 mmHg (intraclass correlation coefficient 0.937), compared with 0.26 ± 1.75 mmHg (0.975) for LithoVue Elite [39]. While these systems aim to reduce operator-dependent variability and minimise exposure to high pressure ranges, clinical outcome data, including comparative data between platforms, remain limited [39]. Nevertheless, these developments collectively represent a significant shift toward pressure-guided endourology, in which IRP becomes an actively monitored and modifiable intraoperative parameter rather than an unmeasured consequence of irrigation. The principal characteristics and supporting evidence for current systems are summarised in Table 2.

### 4.3. Biological and Systemic Implications of Elevated IRP

Elevated IRP is increasingly recognised as a potential risk factor for adverse outcomes following ureteroscopy, particularly infectious complications, although the human evidence is largely observational [15,20]. Pyelovenous and pyelolymphatic backflow permit bacteria, endotoxins, urine, and irrigation fluid to gain systemic access, contributing to postoperative fever, systemic inflammatory response, and urosepsis [13,15,18]. Emerging evidence also suggests that cumulative pressure exposure and transient high-amplitude pressure spikes may be biologically more relevant than peak pressure alone, reinforcing the concept that IRP represents a dynamic physiological exposure rather than a static intraoperative variable [11].

Experimental evidence from porcine models has provided important insight into the biological and histological consequences of elevated IRP during ureteroscopy. Using gadolinium-enhanced magnetic resonance imaging in an in vivo porcine model, Lildal and colleagues demonstrated progressive IRB occurring at increasing IRP levels [18]. Reflux was visualised within the renal cortex at pressures as low as 20 mmHg (27 cmH_2_O) and became increasingly widespread with sustained pressure exposure [18]. Complementing these in vivo observations, Loftus and colleagues demonstrated pressure-dependent parenchymal fluid extravasation and tubular penetration in an ex vivo porcine model simulating URS irrigation conditions [19]. Using India ink-infused saline at sphygmomanometer settings of 50, 100, and 200 mmHg (68, 136 and 272 cmH_2_O), they quantified ink penetration through the renal parenchyma as a percentage of total parenchymal thickness [19]. Without a ureteral access sheath (UAS), higher pressures, particularly 200 mmHg (272 cmH_2_O), resulted in near-complete penetration into cortical tubules and deep parenchymal layers, whereas utilisation of a UAS markedly attenuated this effect, illustrating the protective role of enhanced outflow in mitigating pressure-driven tissue infiltration [19]. Importantly, these histologic findings provide a surrogate model for how irrigation fluid traverses renal structures under elevated IRP and contributes to pyelovenous and pyelolymphatic backflow [18]. Additional experimental studies suggest that excessive IRP impairs renal perfusion, with reductions in renal blood flow observed at higher pressures, indicating a cascade of acute vascular and parenchymal stress beyond mechanical fluid extravasation [13]. Collectively, these findings suggest a continuum of acute injury associated with elevated IRP. At very high pressures, tissue penetration may progress to haemorrhage or forniceal rupture. It has been hypothesised that repeated or sustained exposure could lead to chronic tubular injury or interstitial fibrosis; however, this remains speculative, as no longitudinal animal or human studies have demonstrated persistent histological change, fibrosis or loss of renal function attributable to elevated IRP during ureteroscopy. The acute findings described above also derive from animal and ex vivo models, and their direct applicability to human ureteroscopy remains uncertain.

Human studies increasingly demonstrate an association between elevated intraoperative IRP and adverse postoperative outcomes, although these data are observational [20]. In a multi-institutional analysis of 120 consecutive patients in whom IRP was measured with a pressure guidewire, Croghan and colleagues reported urosepsis in six patients (5%), all after flexible ureteroscopy [20]. Mean IRP during flexible ureteroscopy was higher in patients who developed urosepsis than in controls (81.7 vs. 38.5 mmHg; 111.1 vs. 52.3 cmH_2_O), as was pressure variance [20]. Patients whose admission was prolonged because of postoperative pain had greater pressure variability, although their mean IRP did not differ significantly [20]. The study did not define a safe IRP threshold, and the authors questioned whether the commonly cited limit of 30 mmHg (40 cmH_2_O) is achievable or appropriate in routine practice [20]. These associations do not establish causation and should be regarded as hypothesis-generating rather than as validated clinical targets. Nevertheless, they support the emerging concept that both the magnitude and the variability of IRP exposure may influence perioperative morbidity, and reinforce the importance of pressure-conscious endourological practice.

### 4.4. Patient-Specific Susceptibility to Elevated IRP

The same IRP exposure is unlikely to carry identical biological risk in every patient. A Delphi consensus agreed that baseline and safe pressures are individual to each patient and may depend on patient characteristics [11]. Several patient-level factors appear to modify susceptibility to IRB and its consequences, although most have been characterised in experimental studies or observational cohorts rather than in studies designed to define patient-specific pressure limits.

Infection is the most consistently reported modifier. Positive preoperative urine culture, infected stones and pre-existing obstruction are recognised risk factors for urosepsis after ureteroscopy [9,10,27], and elevated IRP in this setting may facilitate translocation of bacteria and endotoxins via pyelovenous and pyelolymphatic backflow [13,15]. Obstruction and hydronephrosis are associated with papillary thinning and flattening and with altered collecting-system compliance, which may lower the threshold for IRB [13,18]. Papillary morphology is a further determinant: compound or flattened papillae, as seen in medullary sponge kidney and papillary pitting, appear less effective at resisting intrarenal reflux than simple conical papillae [21,26]. Experimental data also indicate that renal resistance to IRB is reduced under hypotensive conditions such as sepsis [10,18,27].

Renal impairment and a solitary kidney reduce physiological reserve, while anatomical factors such as a narrow or tortuous ureter, ureteral stricture, a narrow pelviureteric junction or infundibulum, congenital anomalies and urinary diversion may impede outflow; the Delphi panel identified several of these, together with recurrent urinary tract infection, immunosuppression, diabetes, prolonged stent dwell time and long procedure time, as features of patients at higher risk [11,28]. Direct evidence linking these factors to IRP-related complications is, however, limited. In practice, recognition of these modifiers may justify lower pressure targets, closer pressure monitoring, maximised outflow and early consideration of a staged approach (Section 5.3.4).

## 5. Mitigation Strategies: How We Currently Control Intrarenal Pressure

### 5.1. Ureteral Access Sheaths

UAS reduce IRP by enhancing passive outflow, thereby mitigating IRP rise during irrigation [23,48]. In patients with a nephrostomy in situ, Auge and colleagues recorded mean renal pelvic pressures of 94.4 mmHg (128.3 cmH_2_O) without and 40.6 mmHg (55.2 cmH_2_O) with a UAS during routine stone manipulation [23]. The addition of active suction further augments this effect by enabling controlled evacuation of irrigation fluid and stone debris. Experimental and early clinical studies indicate that suction-assisted UAS, including FANS, can maintain low IRP during flexible ureteroscopy; in 25 patients monitored with a pressure-sensing ureteroscope, median IRP with FANS was 22.0 mmHg (29.9 cmH_2_O), and IRP remained below 40 mmHg (54 cmH_2_O) for 76.2% of procedure time [25,37]. In a two-centre series of 30 patients undergoing FANS-assisted retrograde intrarenal surgery, the median proportion of lithotripsy time with IRP above 30 mmHg (41 cmH_2_O) was 3.3%; this proportion was higher when the sheath tip was positioned at the pelviureteric junction rather than within the renal pelvis, and in staghorn cases [38]. Systems combining real-time IRP feedback with automated control of irrigation and suction have also been evaluated. In a multicentre retrospective feasibility study of 48 patients, a pressure- and temperature-controlled ureteroscope system with a suction UAS maintained IRP below 30 mmHg (41 cmH_2_O) for 99% of lithotripsy time, with no postoperative fever or sepsis reported [46], and in the first-in-human study of an integrated scope-sensor and fluid-management system in 14 patients without active infection, mean IRP was 21.3 ± 9.6 mmHg (29 ± 13 cmH_2_O), with one Clavien–Dindo grade II urinary tract infection [40]. However, these studies were small, largely uncontrolled and primarily evaluated technical efficacy (control of pressure and flow). In contrast, comparative clinical studies of suction-assisted UAS have increasingly reported clinical benefit with respect to infection.

In a meta-analysis of eight comparative studies including 2255 patients, suction UAS were associated with lower rates of postoperative fever (odds ratio [OR] 0.34, 95% confidence interval [CI] 0.24–0.48) and sepsis (OR 0.42, 95% CI 0.23–0.76) than conventional sheaths, together with higher stone-free rates [42]. Meta-analyses restricted to FANS or tip-bendable suction sheaths reported similar reductions in postoperative fever (OR 0.31, 95% CI 0.21–0.46; 10 studies, 2105 patients [43]; risk ratio 0.37, 95% CI 0.25–0.53; 8 studies, 1981 patients [44]), and in a randomised trial of 185 patients with infectious upper urinary tract stones, FANS was associated with lower postoperative inflammatory markers and a lower overall complication rate than a conventional sheath [45]. These data support a clinical benefit of suction-assisted ureteroscopy in reducing infectious complications. However, most contributing studies were retrospective or single-centre, definitions of fever and sepsis varied, and IRP was not measured in most of them. Suction sheaths also improve stone and debris clearance, shorten operative time and evacuate infected fragments, any of which could reduce infectious risk independently of pressure. The reduction in infectious complications associated with suction-assisted ureteroscopy therefore cannot yet be attributed specifically to lower IRP. Evidence that IRP reduction per se, or automated pressure regulation, reduces sepsis, renal injury, readmission or other clinically important outcomes remains limited, and lower IRP should currently be regarded as a physiologically attractive surrogate rather than a proven determinant of improved patient outcomes.

### 5.2. Irrigation Techniques

Irrigation is a principal determinant of IRP during ureteroscopy, with both the delivery method and operator behaviour influencing intrarenal pressure dynamics [13]. Gravity-based irrigation systems generate relatively stable low-pressure flow and are associated with lower IRP profiles, although they may compromise visual clarity [14,29]. In contrast, pressurised irrigation systems and manual hand-pump devices can generate rapid and substantial increases in IRP, particularly when outflow is restricted by ureteral oedema, scope deflection, or working-channel occupancy [30]. Importantly, pressurised and manual irrigation techniques are associated with transient high-amplitude pressure spikes rather than uniform pressure elevation. These brief but repetitive pressure surges are poorly captured by intermittent measurement techniques and are thought to contribute to IRB and tissue injury, although their independent contribution has not been quantified in humans [11]. Operator-dependent variability further compounds this effect, as irrigation intensity is often adjusted reactively in response to bleeding or impaired visibility, creating unpredictable pressure profiles [39]. Emerging irrigation platforms integrating real-time IRP monitoring with automated irrigation-suction modulation are designed to address these challenges, although their clinical benefit remains to be established [39,49].

### 5.3. Procedural Modifications

Beyond devices and irrigation systems, several procedural factors exert a substantial influence on IRP. Optimisation of operative strategy represents a low-cost, universally applicable means of mitigating IRP-related risk. However, most of the strategies below are based on physiological reasoning and expert opinion rather than comparative outcome data; the type of evidence supporting each strategy is summarised in Table 3.

#### 5.3.1. Operative Time

The duration of ureteroscopic procedures is an important determinant of cumulative renal exposure to elevated IRP [20,22]. The concept of an “IRP dose” has been proposed, whereby the biological consequences of elevated pressure are determined not only by peak IRP values but also by the duration and cumulative exposure to pressure over time [20,22]. At present, however, “IRP dose” is an emerging conceptual framework rather than an established quantitative parameter: no standardised or validated metric, such as a pressure–time integral or time above a defined threshold, has been adopted, and no dose–response relationship with clinical outcomes has been demonstrated [11]. Early evidence from percutaneous nephrolithotomy demonstrated an association between postoperative fever and both mean renal pelvic pressure and the accumulated time with renal pelvic pressure ≥ 30 mmHg (41 cmH_2_O), supporting the principle that prolonged pressure exposure may contribute to infectious complications even in the absence of extreme pressure peaks [22].

From a practical perspective, minimising operative duration through efficient stone targeting, judicious basket use, and avoidance of unnecessary calyceal inspection is a physiologically plausible means of reducing cumulative IRP exposure, although its effect on IRP-related complications has not been tested directly [11]. In prolonged or complex procedures, early consideration of staged ureteroscopy may represent a safer alternative to extended periods of sustained high-pressure exposure, particularly in patients with infected stones, impaired renal drainage, or reduced renal compliance; this recommendation is based on expert opinion [11,15].

#### 5.3.2. Scope Size

Ureteroscope diameter influences IRP by affecting outflow around the scope and resistance within the ureteral access sheath, if one is used, or between the scope and the ureter if one is not being used. Larger-calibre scopes, particularly when combined with a narrow ureter or small access sheath, may impair passive egress of irrigation fluid and predispose to pressure elevation [12,14,48,50]. Conversely, smaller-calibre digital ureteroscopes generally facilitate improved outflow and lower baseline IRP, albeit sometimes at the expense of irrigation efficiency or optical performance [12,14,48,50]. Scope selection should therefore balance visual requirements against pressure considerations, particularly in patients with limited renal compliance or increased susceptibility to IRB [12,14].

#### 5.3.3. Fragmentation Strategies

Stone fragmentation technique may influence intrarenal dynamics. Dusting strategies, while often associated with shorter operative times and reduced need for basket extraction, may generate fine particulate debris that transiently obstructs outflow, particularly within access sheaths or when using in-scope suction [14,29,50]. Conversely, fragmentation with active retrieval may prolong operative duration but allow more controlled irrigation and pressure management [50]. The choice of laser settings, fibre size, and fragmentation approach should therefore be individualised, with awareness that frequent scope deflection, prolonged lasering, and working-channel occupancy can contribute to pressure spikes [30,50]. Combining fragmentation strategies with intermittent decompression and controlled irrigation may help mitigate these effects. Comparative data on IRP during dusting versus active retrieval are, however, limited, and these recommendations are based largely on physiological reasoning and expert opinion.

#### 5.3.4. Staged Procedures in High-Risk Patients

In patients at increased risk of IRP-related complications, such as those with infected stones, obstruction, reduced renal compliance, abnormal papillary morphology, or significant comorbidities (Section 4.4), staged ureteroscopy may be considered early in procedural planning [12,13]. Shorter, lower-intensity procedures reduce cumulative pressure exposure and may lower the risk of intrarenal backflow and systemic infection [13,14]. A staged approach allows recovery of renal physiology, resolution of inflammation and optimisation of antimicrobial therapy before definitive stone clearance [12,13,14]. This approach is supported by physiological rationale and expert consensus rather than comparative trials [11]. Importantly, staging should be viewed not as a procedural failure but as a deliberate safety strategy within a pressure-conscious endourological framework.

## 6. The Future: Where Is Intrarenal Pressure Research Going?

Emerging pressure-guided endourological platforms are increasingly moving beyond passive IRP measurement toward integrated fluid management systems capable of dynamically regulating irrigation inflow and suction-assisted outflow. These systems are based on the principle that intrarenal pressure is determined not solely by suction intensity, but by the balance between irrigation inflow, outflow resistance and total flow rate through the collecting system. Recent in vitro work by Glienke and colleagues demonstrated that efficient clearance of stone debris and “sand” fragments during ureteroscopy is primarily dependent on maintaining continuous high flow rates rather than intermittent bursts of suction alone [51]. Using a transparent kidney model, continuous pump-driven flow achieved more efficient debris clearance than conventional pressurised irrigation with intermittent suction, while maintaining IRP within predefined ranges [51].

These findings highlight an important conceptual shift in modern endourology: the goal is not simply maximal suction, but stable low-pressure, high-flow fluid circulation. Conventional systems relying on intermittent manual suction generate transient fluctuations in flow rate and IRP, whereas newer prototype fluid management systems utilise continuous closed-loop regulation of irrigation and suction to maintain stable intrarenal conditions [49,51,52]. Early experimental evaluations suggest that real-time IRP feedback integrated into automated irrigation platforms may permit higher flow rates, improved visibility, and more efficient fragment evacuation without exposing the kidney to sustained hazardous pressure elevations [51]. These are, however, in vitro findings that require clinical confirmation.

Several pressure-monitoring and fluid-management systems are now emerging clinically (Table 2). The i-MIMERsys and Tidor systems integrate continuous pressure monitoring with suction-assisted access, with differing approaches to pressure sensing and fluid evacuation [39]. Pressure-controlled UAS that regulate suction according to measured pressure have been used in clinical series, including in children [47]. More recently, the Asurys™ Fluid Management System (Boston Scientific), which uses the pressure sensor of the LithoVue Elite ureteroscope to adjust irrigation as a surgeon-defined pressure limit is approached, received FDA 510(k) clearance in March 2026 (K251273) [40,41]. Although clinical outcome data remain limited, these technologies collectively suggest a transition toward intelligent fluid ecosystems in which IRP monitoring, irrigation control and suction management function as an integrated procedural safety platform rather than as isolated adjuncts [51].

A key research priority is standardised reporting of IRP. Future studies should report, at a minimum, mean IRP, maximum IRP, time above defined thresholds and pressure variability, and should consider a pressure–time integral, together with the measurement device, sensor location and sampling frequency [11,32]. Standardised metrics would enable comparison across studies, allow the “IRP dose” concept to be tested formally, and facilitate prospective studies linking intraoperative pressure profiles to clinically meaningful outcomes such as sepsis, renal injury and readmission. Studies of patient-specific susceptibility, and of whether pressure-guided ureteroscopy improves outcomes compared with standard practice, are also required.

## 7. Limitations of Current Evidence

Interpretation of the existing IRP literature is constrained by several important limitations. Much of the mechanistic understanding, including most proposed backflow thresholds, derives from animal and ex vivo models, which may not fully replicate human renal physiology or procedural complexity. Human clinical studies are predominantly small and observational, and many evaluate technical efficacy rather than patient outcomes. Conversely, comparative studies reporting fewer infectious complications with suction-assisted sheaths mostly did not measure IRP, so their findings cannot be attributed specifically to pressure reduction. Pressure measurement techniques, sensor locations and reporting units (mmHg or cmH_2_O) vary widely across studies, limiting comparability and reproducibility, and pressure measured at a single site may not reflect pressure elsewhere in the collecting system. Long-term human outcome data linking intraoperative IRP profiles to renal function or oncological endpoints are sparse, and no longitudinal animal or human studies have shown that IRP exposure during ureteroscopy causes persistent histological change or renal fibrosis. Isolating the independent effects of pressure from confounding variables such as infection, operative duration, irrigation volume and instrumentation remains methodologically challenging. Finally, as a narrative review, this article is subject to potential selection bias in the identification and interpretation of studies.

## 8. Conclusions

Despite growing recognition of IRP as a clinically relevant and modifiable factor during ureteroscopy, definitive clinical guidelines remain limited by the lack of large prospective human studies. IRP is not routinely monitored during ureteroscopy in current practice. However, recent technological advances have made real-time measurement feasible with scope-integrated and suction-assisted systems, supporting a transition from passive pressure estimation toward pressure-guided endourology. Emerging evidence suggests that cumulative pressure exposure, pressure variability and transient spikes may be more clinically relevant than peak IRP alone [11,20]. Intelligent fluid-management platforms integrating pressure monitoring with automated irrigation and suction regulation may therefore enable safer maintenance of high-flow, low-pressure intrarenal environments during ureteroscopy while reducing operator-dependent variability, although their effect on clinical outcomes has yet to be demonstrated. Suction-assisted sheaths are associated with fewer infectious complications, but whether this reflects lower IRP or other effects, such as improved stone and debris clearance, remains to be established. These observations underscore the need to move beyond static pressure thresholds toward a more nuanced, pressure-guided approach to endourology, supported by standardised IRP reporting and prospective human studies that link intraoperative pressure profiles to meaningful clinical outcomes.

## Figures and Tables

**Table 1:** Reported intrarenal pressure values grouped by the nature of the pressure value: (A) experimental backflow thresholds; (B) irrigation pressure settings; (C) observational clinical measurements; (D) physiological reference values and expert consensus. Experimental thresholds describe the onset of intrarenal backflow in animal or ex vivo models and are not validated human safety limits.

Pressure, mmHg (cmH_2_O)	Reported Finding	Model or Population	Nature of Pressure Value and Type of Evidence	Refs
A. Experimental intrarenal backflow thresholds (animal and ex vivo models)				
20–30 (27–41)	Onset of pyelotubular backflow	Animal models	Measured IRP at backflow onset; experimental	[15,21]
30–50 (41–68)	Pyelovenous backflow	Animal models	Measured IRP at backflow onset; experimental	[15]
>70 (>95)	Pyelointerstitial backflow	Animal models	Measured IRP at backflow onset; experimental	[15]
20–70 (27–95), sustained 30–60 min	Progressive cortical IRB on gadolinium-enhanced MRI, detectable from 20 mmHg	Porcine, in vivo	Controlled, measured IRP; experimental	[18]
B. Irrigation pressure settings (pressure applied at the irrigation source)				
50–200 (68–272), irrigation setting	Pressure-dependent parenchymal ink penetration, attenuated by a UAS	Porcine, ex vivo	Applied irrigation pressure; IRP not measured; experimental	[19]
C. Observational clinical measurements of intraoperative pressure (human)				
Mean RPP ≥ 20 (≥27); accumulated time with RPP ≥ 30 (≥41)	Associated with postoperative fever after mini-PCNL	Human, 80 patients	Measured RPP; clinical association (observational)	[22]
Mean 81.7 vs. 38.5 (111.1 vs. 52.3)	Higher mean IRP and pressure variance in patients developing urosepsis (6/120); no safe threshold defined	Human, 120 patients, URS	Measured IRP (guidewire); clinical association (observational)	[20]
Mean 94.4 without vs. 40.6 with UAS (128.3 vs. 55.2)	Lower renal pelvic pressure with a UAS during stone manipulation	Human, URS with nephrostomy in situ	Measured RPP (nephrostomy); observational	[23]
Mean 30.8 calyceal vs. 17.9 pelvic, with UAS (41.9 vs. 24.3)	Intracalyceal pressure frequently differs from renal pelvic pressure	Human, 8 patients (45 calyces), URS	Measured calyceal pressure (guidewire); observational	[24]
Median 22.0 (29.9); <40 (<54) for 76.2% of procedure time	IRP during FANS-assisted flexible URS	Human, 25 patients	Measured IRP (scope tip); observational	[25]
D. Physiological reference values and expert consensus				
0–15 (0–20)	Physiological renal pelvic pressure	Physiological measurements; consensus definition (~15 cmH_2_O)	Physiological reference; expert consensus	[11,15]
~29 (40)	Level at which initial concern begins; no evidence-based threshold agreed	Delphi panel of endourologists	Expert consensus; no measurement	[11]

FANS, flexible and navigable suction ureteral access sheath; IRB, intrarenal backflow; IRP, intrarenal pressure; PCNL, percutaneous nephrolithotomy; RPP, renal pelvic pressure; UAS, ureteral access sheath; URS, ureteroscopy. Values converted at 1 mmHg ≈ 1.36 cmH_2_O. Irrigation pressure settings are pressures applied at the irrigation source and are not equivalent to measured IRP. Clinical measurements were obtained with different devices and at different sensor locations and are not directly comparable.

**Table 2:** Comparison of current intrarenal pressure monitoring and fluid-management systems.

Device or System (Manufacturer)	Pressure-Sensing Location	Irrigation; Suction	Closed-Loop Regulation	Type of Evidence (Number of Human Patients Studied)	Regulatory Status	Demonstrated Clinical Outcomes
LithoVue Elite ureteroscope (Boston Scientific, Marlborough, MA, USA)	Ureteroscope tip	Gravity or pump irrigation; no integrated suction (compatible with suction UAS)	No (monitoring only)	Porcine experimental study [30]; retrospective first-in-human clinical series (46) [34]; observational series with FANS (25 [25]; 30 [38])	FDA 510(k) K221515 (2023) [33]	Feasibility of continuous IRP monitoring; no outcome benefit demonstrated
Asurys Fluid Management System (Boston Scientific, Marlborough, MA, USA)	Uses LithoVue Elite tip sensor	Automated pump irrigation adjusted or paused as a surgeon-set limit is approached; suction not specified in cited sources	Yes	Prospective first-in-human observational study (14) [40]	FDA 510(k) K251273 (March 2026) [41]	Mean IRP 21.3 mmHg (29 cmH_2_O); one Clavien–Dindo grade II urinary tract infection (uncontrolled)
i-MIMERsys (ZSR Biomedical Technology Co., Ltd., Dongguan, Guangdong, China)	Sheath-based sensor	Irrigation and suction autoregulation via FANS	Yes	In vitro accuracy study only (no human patients) [39]	Not stated in cited sources	None reported
Tidor (YiGao Medical Technology Co., Ltd., Hangzhou, Zhejiang, China)	Sheath-based sensor	Suction-assisted outflow	Not specified in cited sources	In vitro accuracy study only (no human patients) [39]	Not stated in cited sources	None reported
Suction UAS and FANS without integrated pressure sensing (various manufacturers)	None (IRP not measured in most comparative studies)	Conventional irrigation; continuous or surgeon-controlled suction via the sheath	No	Comparative cohort studies and RCTs pooled in meta-analyses (2255; 2105; 1981) [42,43,44]; RCT in infectious stones (185) [45]	Varies by device; not reviewed	Lower postoperative fever and sepsis than conventional UAS; benefit not shown to be attributable to IRP reduction
Pressure-sensing FANS [37]	Sheath tip	Conventional irrigation; FANS suction	No	Preclinical animal study and pilot clinical study (100, compared with conventional FANS) [37]	Not stated in cited sources	IRP maintained at ≤30 mmHg (41 cmH_2_O) with higher irrigation flow; complication rates similar to conventional FANS
Pressure- and temperature-controlled ureteroscope system with suction UAS [46]	Ureteroscope	Pump irrigation; suction UAS	Yes	Multicentre retrospective feasibility study (48) [46]	Not stated in cited sources	IRP < 30 mmHg (41 cmH_2_O) for 99% of lithotripsy time; no fever or sepsis (uncontrolled)
Intelligent pressure-controlled UAS	Sheath	Irrigation through ureteroscope; pressure-regulated suction via sheath	Yes	Clinical series, including 21 children [47]	Not stated in cited sources	Stone-free rate 85.7% at 1 month; no Clavien–Dindo grade ≥ III complications (uncontrolled)
Intravascular pressure guidewires (e.g., PressureWire, Verrata, COMET II)	Guidewire tip within collecting system	Independent of irrigation and suction	No	Pilot and observational clinical studies (20 [35]; 8 [24]; 120 [20])	Vascular devices; urological use in research settings	Measurement tool; higher IRP associated with urosepsis (observational) [20]

Regulatory status is reported only where verified from FDA records; FDA 510(k) clearance indicates substantial equivalence to a predicate device and is not an approval based on clinical outcome data. Numbers in parentheses are human patients studied; meta-analyses include overlapping study populations. FANS, flexible and navigable suction ureteral access sheath; IRP, intrarenal pressure; RCT, randomised controlled trial; UAS, ureteral access sheath.

**Table 3:** Strategies to mitigate intrarenal pressure, classified by the type of supporting evidence.

Strategy	Proposed Mechanism	Type of Evidence	Refs
Conventional UAS	Increases passive outflow	Evidence-based (experimental and clinical IRP measurements)	[23,30,48]
Suction UAS, including FANS	Active outflow and debris evacuation	Evidence-based for IRP reduction; associated with fewer infectious complications in comparative studies and meta-analyses, but benefit not shown to be attributable to IRP reduction	[25,37,38,42,43,44,45]
Automated pressure-controlled irrigation and suction	Closed-loop regulation of inflow and outflow	Evidence-based for technical efficacy in pilot studies; patient benefit not established	[40,46]
Gravity or low-pressure irrigation; avoiding uncontrolled hand-pump boluses	Limits inflow pressure and pressure spikes	Evidence-based (experimental and clinical IRP measurements)	[14,29,30]
Smaller scope or favourable scope-to-sheath ratio	Improves outflow around the scope	Evidence-based (experimental flow and pressure data)	[29,31,48]
Real-time IRP monitoring	Allows recognition and correction of pressure elevation	Physiologically plausible; supported by expert consensus	[11,32]
Limiting operative time	Reduces cumulative pressure exposure	Physiologically plausible; indirect clinical association	[20,22]
Avoiding unnecessary calyceal inspection	Shortens duration of pressure exposure	Expert recommendation	[11]
Choice of dusting versus active retrieval	Balances debris-related outflow obstruction against operative duration	Expert recommendation	[50]
Intermittent decompression	Allows pressure to fall between periods of irrigation	Physiologically plausible	[14]
Staged ureteroscopy in high-risk patients	Limits cumulative exposure; allows treatment of infection	Expert recommendation	[11,15]

Evidence-based: supported by experimental and/or clinical studies measuring IRP; physiologically plausible: mechanistic rationale with indirect or limited supporting data; expert recommendation: based predominantly on expert opinion or consensus. Although suction-assisted sheaths are associated with fewer infectious complications, no strategy has yet been shown in comparative studies to reduce complications specifically through IRP reduction. FANS, flexible and navigable suction ureteral access sheath; IRP, intrarenal pressure; UAS, ureteral access sheath.

## Data Availability

No new data were created or analyzed in this study. Data sharing is not applicable to this article.
